# Pica, avoidant restrictive food intake disorder and rumination disorder in children and adolescents with autism spectrum disorder: A systematic review

**DOI:** 10.1186/s40337-026-01772-x

**Published:** 2026-09-14

**Authors:** Gunhild Hodt, Gro Janne Henningsen Wergeland, Ola Skjeldal, Jørn Isaksen, Mads Holten-Andersen, Vesna Bryn, Maj-Britt Posserud

**Affiliations:** 1 Department of Child and Adolescent Psychiatry, Innlandet Hospital Trust, Lillehammer, Norway; 2 Institute of Clinical Medicine, Faculty of Medicine, University of Oslo, Oslo, Norway; 3 Department of Clinical Medicine, Faculty of Medicine, University of Bergen, Bergen, Norway; 4 Department of Child and Adolescent Psychiatry, Division of Psychiatry, Haukeland University Hospital, Bergen, Norway; 5 Sahlgrenska University Hospital, University of Gothenburg, Gothenburg, Sweden; 6 University of Inland, Lillehammer, Norway; 7 Department of Pediatric and Adolescent Medicine, Innlandet Hospital Trust, Lillehammer, Norway

**Keywords:** Pica, Rumination syndrome, Rumination disorder, Avoidant restrictive food intake disorder, ARFID, Children, Autism spectrum disorder, ASD

## Abstract

**Background:**

Children with autism spectrum disorder (ASD) have an increased risk of co-occurring eating disorders such as pica, avoidant restrictive food intake disorder (ARFID) and rumination disorder (RD). The aim of the current systematic review was to synthesize and appraise the existing literature of pica, ARFID and RD in children with ASD regarding occurrence, diagnostic assessment, clinical pathways, intervention and the quantity and design of the included studies.

**Methods:**

We conducted a systematic review in accordance with the PRISMA guidelines. MEDLINE, Embase, Cochrane, CINAHL and PsycINFO were searched up to January 12th, 2026. Inclusion criteria were peer reviewed studies in English, age < 18 years, sample of n ≥3 and diagnoses according to DSM-IV/DSM-5, or ICD-9/ICD-10 or the upcoming ICD-11. Eating disorders describing ARFID symptoms or ARFID phenotypes without a formal diagnosis were excluded.

**Results:**

The search strategy resulted in a total of 8,678 potentially eligible articles, of which 23 fulfilled inclusion criteria. Ten studies examined comorbid ASD and ARFID, twelve ASD and pica and one ASD and rumination disorder. The included studies indicated an increased risk of pica and ARFID in children with ASD, with potentially severe adverse outcomes appearing responsive to early detection and intervention. RD may still be underrecognized and understudied in ASD.

**Conclusions:**

This study confirms a substantial co-occurrence of ASD and eating disorders in which concerns regarding weight and body shape are not the primary drivers. However, there is a scarcity of studies, and the studies have small cohorts and lack standardized diagnostic assessment and treatment. Early detection of these eating disorders may improve clinical outcome.

**Supplementary Information:**

The online version contains supplementary material available at https://doi.org/10.1186/s40337-026-01772-x.

## Abstract (plain language summary)

Children with autism spectrum disorder (ASD) are more likely to experience feeding and eating difficulties than children without ASD. Some of these difficulties may meet criteria for eating disorders such as pica, avoidant/restrictive food intake disorder (ARFID), and rumination disorder. These eating disorders are not driven by body shape and weight concerns, but can still affect nutrition, growth, physical health, and daily functioning.

This systematic review examined published research on pica, ARFID, and rumination disorder in children with ASD. We identified 23 studies that met inclusion criteria. Most studies focused on pica or ARFID and only one on rumination disorder. Many studies included small numbers of participants and used different methods for diagnosis and assessment, making comparisons difficult. There was also limited research on treatment approaches and long-term outcomes.

Although research is still limited, the findings suggest that early detection and treatment may improve outcomes for children with ASD and these eating disorders. Greater awareness of their coexistence may help support earlier recognition and referral. However, more research is needed to fill the knowledge gap on children with ASD and co-occurring pica, ARFID and rumination disorder.

**Supplementary Information:**

The online version contains supplementary material available at https://doi.org/10.1186/s40337-026-01772-x.

## Introduction

Autism spectrum disorder (ASD) is characterized by persistent deficits in social communication and interaction across multiple contexts, and restricted and repetitive patterns of behaviour, interests or activities [1]. The disorder is hypothesized to influence food intake in children with autism; however, food related difficulties may also be associated with co-occurring anxiety, the social demands of eating or symptoms of hyperactivity and inattention across neurodevelopmental conditions [2, 3]. Previous reviews have reported that as many as 50–90% of children with ASD have feeding and eating problems [4, 5] potentially resulting in weight loss, failure to grow, overeating, overweight, psychosocial problems and even fatal outcomes [5]. Subsequent reviews report that there is an increased risk of eating disorders like anorexia nervosa [6, 7], but also of eating disorders not motivated by concerns about body shape and weight [1] such as pica, avoidant restrictive food intake disorder (ARFID) and rumination disorders (RD). Existing literature on the association of ASD and anorexia nervosa, originally proposed by Gillberg in 1983 [8], is quite extensive. Subsequent research, including a recent systematic review and meta-analysis, has demonstrated an over-representative of autistic traits and ASD among individuals with anorexia nervosa [9] and ARFID [10] This established association has prompted increasing interests to further investigate how feeding and eating disorders, like pica, ARFID and RD, are associated with ASD.

## Pica, ARFID and RD in ASD

Pica is characterized by the persistent consumption of non-nutritive, non-food substances, such as plastic, cloth, paper, dirt, soap, rocks, paint and cigarette butts [11]. The condition may result in serious and possibly fatal medical complications such as poisoning, choking, infections, perforation or inedible items stuck in the gastrointestinal tract or stomach [12, 13]. Pica is not exclusively associated with ASD, although the prevalence appears to be higher among children with ASD than in the general population. Fields et al., [14] reported that pica was present in 14% in preschool-aged children with ASD without intellectual disabilities, compared with 3,5% in the general population of children [14]. This aligns with findings from Lesinskienė et al., [15] who observed that 3,7% of preschool children engaged in the consumption of non-nutritious, non-food substances. In their study, children with increased sensory sensitivity traits ate significantly more non-nutritious, non-food substances than their peers.

ARFID was first presented in the DSM-5 [1] and describes a restrictive eating behaviour that can be driven by low interest in eating, food avoidance based on the sensory characteristics of food and/ or fear of the aversive consequences of eating (e.g. chocking phobia), the so-called ARFID drivers. According to the diagnostic criteria of ARFID, the avoidance and restriction of intake of food should not be caused by disturbances or concerns of body image and weight. The clinical presentation is heterogeneous, often leading to impairment manifested as malnutrition, psychosocial dysfunction or dependence of supplements or tube feeding to meet nutritional needs. Importantly, the weight can be normal, under, or above the expected weight for age and height (Bourne et al., 2021; [16–18]). A scoping review reports that the co-occurrence of ARFID-like presentation in ASD is common [19]. In their meta-analysis Sader and colleagues [20] found the ASD prevalence to be 16,3% in participants with ARFID, and the ARFID prevalence to be 11,4% in the ASD group [20]. However, until recently, research exploring the association between ASD and ARFID has been limited [10].

Rumination disorder or rumination syndrome is characterized by effortless, repetitive regurgitation of recently ingested food, followed by either rechewing, reswallowing or spitting the food out [1]. Rumination occurs when the abdominal wall contracts, forcing the gastric contents back to the oesophagus and mouth [21]. The rumination disorder is often underrecognized and may be misunderstood as gastroesophageal reflux disease (GERD), bulimia or anorexia nervosa [22]. Reported prevalence of RD in children varies depending on type of study, population and diagnostic criteria. In a systematic review and meta-analysis by Haworth et al., [21] the pooled prevalence in children was 0,4% according to the Rome IV criteria [23], 1.0% according to the Rome III criteria [24] and 2.1% when self-reported [21]. The prevalence was reported to be 2.0% (Rome III) in a paediatric functional abdominal pain population [25] and 5.1% in a population-based sample [26]. However, knowledge is scarce regarding the prevalence of rumination disorder in children with ASD.

Although recent reviews have addressed pica, ARFID and RD in children with ASD, several limitations necessitate the conduction of an updated synthesis of the evidence. In a scoping review, evidence was synthesized on the nature, consequences and treatment of ARFID-like symptoms in young people with ASD [19]. However, only two of the included studies specifically reported on individuals with concurrent diagnosis of ARFID and ASD, including a single case study [27] and a medical record review [28]. In the prevalence-based meta-analysis Sader et al., [20] included individuals with a diagnosis of both ARFID and ASD, investigated the association between autistic traits and anorexia nervosa and ARFID, but did not distinguish by age when reporting prevalence, and did not examine assessment, outcome or treatment. Existing systematic reviews on pica [29] and rumination disorder [21] have not included co-occurring ASD specifically. Moline et al., [29] examined the effectiveness of behavioural treatments for pica in children in general and reported ASD as a comorbid condition.

In sum, there has been a growing body of research on the topic of eating and feeding disorders in children with ASD, however, studies examining ASD and comorbid pica, ARFID and/or rumination according to standard classification systems remains scarce. Furthermore, the lack of standardized data collection methods and variation in diagnostic criteria, cut-offs, and assessment measures may limit the comparability and synthesis of research findings on the co-occurrence of ASD and eating disorders. This may contribute to inconsistent prevalence estimates, difficulties in identifying comorbid presentations, and challenges in developing evidence-based assessment and treatment approach. These gaps highlight the need for a systematic synthesis of the available evidence. Therefore, the aim of the current study was to systematically review the occurrence, diagnostic assessment, clinical pathways, and interventions for pica, ARFID and rumination disorder in children with ASD, as well as the quantity and design of the included studies.

## Materials and methods

The systematic review was conducted according to the Preferred Reporting Items for Systematic-reviews and Meta-Analysis (PRISMA) statement [1, 30]. A comprehensive protocol was developed a priori to guide the research process. We initially attempted to register the protocol in PROSPERO prior to study initiation. However, due to technicalities, the registration process stalled. Because of the rapid progression of our timeline and the clinical relevance of the data, the review was not registered because retrospective registration is inappropriate according to PROSPERO guidelines.

The search was structured around a PEO framework in the following way: Children and adolescents under 18 years of age constituted the population (P), ASD represented the exposure (E), and the outcomes (O) included ARFID, pica and RD. This framework informed the selection of search term and eligibility criteria.

### Literature search

Relevant studies were identified through a systematic and comprehensive literature search of the electronic databases Ovid MEDLINE, Embase, Cochrane, CINAHL and PsycINFO, from 1990 - January 21st, 2025. An updated search was done on January 12th, 2026.

The list of search terms was generated by the authors in collaboration with a university librarian who also conducted the database searches. For the search we used both subheadings and free text and words. Search terms included “feeding disorder*”, “eating disorder*”, “malnutrition”, “pica”, “Avoidant Restrictive Food Intake disorder”, “ARFID”, “food neophobia*”, “food selectivity”, “fussy eating”, “Rumination disorder*”, “Rumination Syndrome”, terms related to ASD including “asperger*”, “autism*”, “PDD”, “autistic*” AND “all child (0–18 years)”. The search was evaluated through the PRESS Guideline – Search Submission and Peer Review Assessment [31]. For the full search strategy see supplementary information [S1]. In addition, the reference lists of the most recent systematic reviews and meta-analyses were screened for relevant publications potentially missed in the search.

Rayyan was used as software system for recording decisions [32]. Four pairs of authors (MP-GJW, GH-MH, OS-JI, GH-VB) screened the title and abstract independently to consider whether a study should result in further reading. Disagreements were discussed and resolved by consensus primarily in pairs, and if needed or when doubts, with the other author pairs. Full-text articles were retrieved if there was any indication that the study included children with ASD and pica, ARFID and/or rumination disorder.

### Inclusion criteria

To be included the study had to:

1. Be a peer reviewed article published, or in press, in an English language journal.
2. Have participants diagnosed with ASD according to DSM IV or later or ICD 9 or later.
3. Have a diagnosis of ARFID, pica and/or RD according to the DSM IV or later or ICD 9 or later.
4. Include a sample consisting of at least three participants.
5. Include children with mean age < 18.

### Exclusion criteria

1. All children in the sample fulfilled a diagnosis of an additional comorbidity when the additional comorbidity by itself challenges eating and feeding, e.g. syndromes or conditions.
2. Secondary analysis of already published data.
3. All included patients are part of a pharmacological medication study evaluating effect of medications.

### Categorization of studies/data extraction

Data were extracted from the articles, including study design, assessment of diagnoses, participant demographics and clinical characteristics of the participants. The data to be extracted were agreed upon a priori and were recorded in a spreadsheet including the variables of interest. The data extraction was done independently in the pairs. A formal quality assessment was not possible due to study heterogeneity; however, methodological limitations were evaluated in the discussion.

## Results

### Study selection

**Fig. 1:**
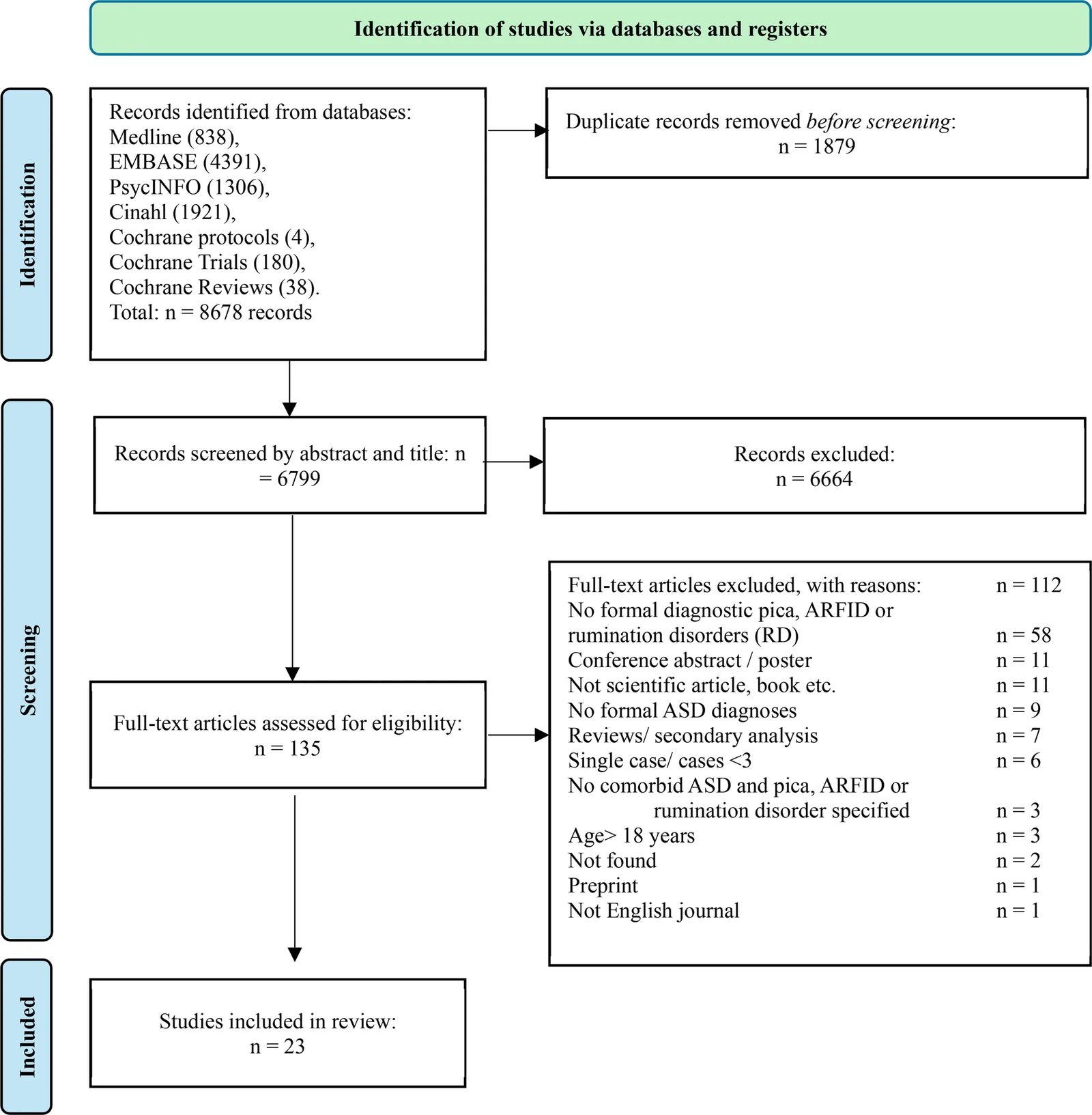
PRISMA 2020 flow diagram of the study selection process

The search resulted in a total of 8678 articles. A total of 23 studies were included (Fig. 1).

### Study characteristics

The included studies varied across countries, clinical settings, and sample sizes. Twelve studies examined pica in ASD, ten examined ARFID in ASD, and only one examined RD in ASD (Tables 1, 2 and 3).

**Table 1:** Study characteristics of included studies: ASD and PICA

Study	Design	Country	Setting	Sample size:	Sample age (years)	Diagnostic assessment ED	Frequency: % pica in ASD (% pica in other)	Outcomes/ Further findings
Adams et al., [33]	Case-control	USA	Members of a autism society	ASD: 51 CC: 40	3–15 Mean 7.1 (SD 3.0) in ASD	M: reports. Hair samples compared	32 (0 in CC)	Pica + ASD: Sodium, chromium and sulphur levels were low. Strontium and copper elevated. Toxic metal: normal Chromium much lower in pica + ASD vs. ASD (*p* = 0.004) or CC (*p* = 0.002).
Ahumada et al., [34]	Cross-sectional	Chile	Community based	ASD: 72	2–12	P: Questionnaires [43, 44, 63]	16,7	Xylophagia 4.2% Foliophagy 4.2% Food selectivity: 91.7%
Bicer et al., [35]	Cross-sectional observational study	Turkey	Four autism rehabilitation centres	ASD:115	4–18	3 day food records. Questions by authors. FHQ. Blood. Nutrition intake calculated	10,4	ASD: 58.5% overweight/obese and 11% severely thin/thin. ASD + pica: 8% and 2.4% respectively. Food selectivity: 30%
Brown et al., [36]	Retrospective cross-sectional study	UK	Community-based autism assessment clinics	ASD:102	Median 4.5	Medical records. Blood	13.7	BLC ≥ 0.24 µmol/L in 12% with no pica and 23% with pica. Advice BLC screening in ASD.
Call et al., [12]	Clinical intervention	USA	Intensive day - treatment clinic	Pica: 11		Record review. Observations.	8/11 pica + ASD Of which 5/8 also ID, 1/8 also ADHD, 1/8 also ADHD/ID	Behavioural interventions are highly effective treatment for pica.
Emond et al., [37]	Longitudinal prospective	UK	Population based.	ASD: 79 CC:1290	5 check-points age 0.5–4.5	P: FFQ, BAMBI. Somatic examination. Nutrition intake calculated *	12.3 and 12.5 (2.3 and 0.7 in CC) at 3.2 and 4.5 years respectively	Children with ASD showed feeding symptoms from infancy.
Fields et al., [14]	Multisite case-control	USA	Population based Multi-ethnical	ASD: 1244 Other DD:1593 CC:1487	2.5–5.7 Median age: 4.6	CBCL P: health-history forms.	22.7 (8.3 in DD) (3.4 in CC)	ASD with pica was associated with vomiting (*p* < 0.01), diarrhoea (*p* < 0,05) and loose stool (*p* < 0.05), not constipation or pain on stooling.
Fields et al., [38]	Multisite case-control	USA	Population based Multi-ethnical	ASD: 1426 DD:1735 CC:1578	2–5	CBCL M: Structured telephone interview.	23.2 (8.4 in DD) (28.1 in ASD + ID) (3.5 in CC) (14 ASD no ID)	Pica is common in preschool-aged children with ASD.
Frank-Crawford et al., [39]	Consecutive controlled case series study. Retrospective	USA	In- and outpatient clinic specialised in challenging behaviour	Pica: 33, in which ASD: 31	6–19 Mean age: 11.2	Medical records		Behaviour treatment: 90% reduction in pica after final treatment. Caregiver involvement.
Mayes et al., [40]	Cross-sectional observational	USA	National standardisation sample + Child psychiatry diagnostic clinic	ASD: 1462 ADHD/ other: 327 CC: 313	1–18 Mean age: 7.3	P: CASD	12	Symptoms of pica and pocketing food only reported in ASD children. Atypical eating: 70.4% in ASD vs. 13.1% in other disorders vs. 4.8% in CC.
Papini et al., [41]	Longitudinal prospective	UK	Population based	Pica:312	5 check-points age (3–9.6)	P: Questions of pica, FFQ, BAMBI Routine preschool aged child health follow-up*	10.7–13.6% in ASD.	Pica behaviour and BMI not associated. Significant association between pica and autism found.
Prabhakaran et al., [42]	Cross-sectional analysis	South India.	Autism clinic	ASD: 96	3–18	Semi-structured proforma by authors, GISSI-17	36,5% in ASD	Sensory patterns predictive of pica.

*C* children, *CC* control children, *M* mothers, *P* parents, *ADHD* Attention Deficit Hyperactive Disorder, *ASD* autism spectrum disorder,* BAMBI* Brief Autism Mealtime Behaviour Inventory, *BLC* blood lead concentration, *BMI* body mass index, *CBCL* Child Behaviour Checklist, *CASD* Checklist forautism spectrum disorder (feeding problems1/30 items with five subitems including eating inedibles), *DD* developmental delay, *ED* eating disorder, *FFQ* Food Frequency Questionnaire, *FHQ* Feeding History Questionnaire by The Children´s Hospital of Philadelphia (2013), *GISSI-*17 Gastrointestinal symptom inventory for ASD, *ID* intellectual disability

*All questionnaires at ALSPAC website

**Table 2:** Study characteristics of included studies: ASD and ARFID

Study	Design	Country	Setting	Sample size:	Sample age (years)	Diagnostic assessment of eating disorders	Frequency: % ARFID and ASD (other)	Outcomes/ Further findings
Bourke et al., [45]	Case series Medical records in retrospect	UK	Tertiary hospital care centre	(ARFID + ASD): 3	6, 8 and 16	Diet anamneses Blood	3/5: All 5 with visual impairment + optic atrophy	Optical canal hyperostosis with compressive optic neuropathy secondary to Vitamin A deficiency. Irreversible despite Vitamin A supplement.
Calisan Kinter et al., [46]	Case control	Turkey	Outpatient clinic	ASD: 37 (ARFID + ASD): 37 CC: 37	4–10	CEBQ SP		Significant lower weight, height and BMI in ASD + ARFID vs. ASD and vs. CC. FSIQ: significant higher ASD vs. ASD + ARFID. Oral processing scores significantly predicted ARFID comorbid to ASD. SRS higher in ASD + ARFID.
Datta et al., (2025)	Chart review Retrospect Intervention study	USA	Outpatient ED, University clinic	ARFID: 1. 39 2. 40	1. <13 2. 13–18	DSM-5 criteria-based assessment by physician and dietician	1. 3/39 2. 4/40	FBT + ARFID: FBT-ARFID is feasible for treating, retaining and obtaining outcomes in adolescents, as well as in children. Weight gain alone insufficient parameter of recovery.
Farag et al., [47]	Multisite control	UK	3rd multi-disciplinary feeding clinic	1.ARFID 263 2. No -ARFID: 273	Mean age 6.8 SD 3.4	DSM-5 criteria and consensus.	1. 58,3 2. ASD in NoARFID 41.7	ARFID in young children may indicate ASD. ASD + ARFID: 61% with inadequate nutrition, of whom 54.9% was inadequately supplemented. 6.3% dependent on enteral supplements. Most deficiencies are correctible.
Islam et al., [48]	Case series	USA	Multi-disciplinary clinics	(ARFID + ASD): 4	12, 13, 14 and 17	Diagnostic interview Clinical assessments Blood Radiology Meal observations		BMI: ¾ normal to obese. Dietary screening identified health risks. Compression fractures: 2/4 and Rickets: 2/4 due to Vitamin D deficiency. Vitamin supplement needed. In one patient: biphosphate therapy.
Lien et al., [49]	Clinical prospective case series. Intervention	Denmark	Specialized outpatient ED clinic	(ARFID + under-weight): 33	6.3–18	PARDI Somatic evaluation List: never-always-sometimes	(ASD + ARFID + underweight): 42	FBT-ARFID: Stable weight 21%. Median weight gain significant at group level (with/without ASD).
Nygren et al., [50]	Prospective longitudinal	Sweden	Multiethnic low resource area	ASD: 46	preschool	Broad multidisciplinary medical evaluation DSM-5 ARFID criteria	(ASD + ARFID): 28 (Feeding problems + ASD): 35/46	ARFID criteria: A1) BMI in SD ≤-2: 12, A2) Iron deficiency in 3 of 10 with significant nutritional deficiency, A3) oral nutritional supplement: 9 A4) psychosocial impairment: 12. Coexisting medical feeding issues: 5
Sanchez -Cerezo et al., (2024)	Observation -surveillance	UK and Republic of Ireland	Secondary Clinical care	ARFID: 319	5–18	Questionnaire by authors DSM-5 ARFID criteria	(ARFID + ASD): 44	SDS BMI: ASD-noARFID > ASD-ARFID (significant). Height and weight SDS: ASD-ARFID < ASD-no-ARFID (significant). Significant more ASD in “combined” and “sensory” subtypes than in “fear” and “lack of interest” subtypes.
Tanaka et al., [51]	Retrospective study	Japan	University hospital	ARFID: 1. 33 2. 15	4–16 Median age 9.5	Clinical examination DSM-5-TR ARFID criteria	ASD in ARFID according to prognoses 1. Good: 21 2. Poor: 60	ASD not initially diagnosed in more than half of the ARFID patients with comorbid ASD.
Watts et al., [52]	Cross-sectional	UK	Maudsley centre for ED, outpatient clinic	ARFID: 261	9.2–15.8 Median age 12.7	P: PARDI-AR-Q Clinical examination DSM-5ARFID criteria	(ARFID + ASD): 28	ARFID is multi-faceted and heterogenous. Clinical presentations largely independent of sex, ASD and anxiety traits. “Sensory sensitivities” and “lack of interest” more common in ARFID + ASD than in ARFID-noASD.

*CC* control children, *CEBQ* Child eating behaviour questionnaire, *ED* Eating disorder, *DSM*-5: Diagnostic and Statistical Manual of Mental disorders 5th edition, *FSIQ* Full Scale Intelligence Quotient, *P* parents, *PARDI* Pica, ARFID and Rumination disorder interview, *PARDI*-*AR*-*Q* Pica, ARFID and Rumination disorder interview ARFID Questionnaire, *SP* Sensory Profile Caregiver Questionnaire

**Table 3:** Study characteristics of included studies: ASD and Rumination disorder

Study	Design	Country	Setting	Sample size:	Sample age (years)	Diagnostic assessment of eating disorders	Frequency: % RS in ASD (other)	Outcomes/ Further findings
Akbal Bagci et al., [53]	Cross-sectional observation study	Turkey	Outpatient clinic. Child psychiatry	ASD: 97	3–12 Median age 6	DSM-5 QPGS-RIII	3.1 (Functional GIS: 38.1)	Evaluate children with ASD for GIS and sleep problems, especially if behavioural challenges.

*GIS* gastrointestinal symptoms, *QPGS-RIII* Questionnaire on Paediatric Gastrointestinal Symptoms, Rome III criteria, *RS* rumination syndrome

### Pica and ASD

**Fig. 2:**
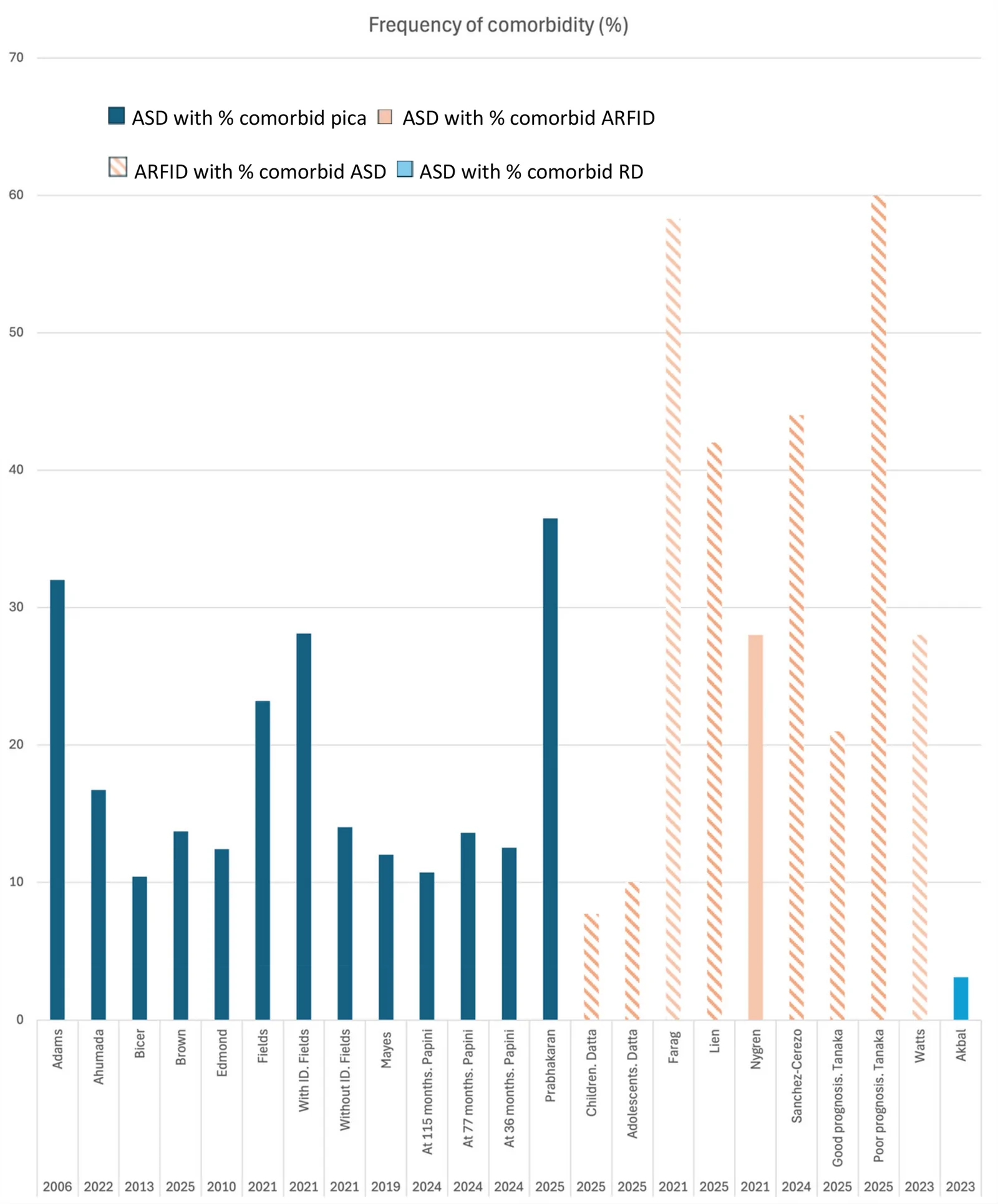
Studies reporting on frequencies of eating disorders comorbid with ASD

Twelve studies examining pica and ASD were identified (Table 1). The studies reported on a wide variance in themes and foci. In the nine studies reporting on frequency, ASD was diagnosed before pica. The frequency of pica comorbid to ASD (Fig. 2) ranged from 10,4% [35] to 36,5% [42]. The highest prevalence was reported in a study investigating gastrointestinal symptoms in ASD. While sensory patterns were found to predict pica, no association between pica and BMI was identified [35, 41]. In the one treatment study that started out with a selected group of children with pica, only two out of 33 children did not have ASD in addition to pica [39]. One study analysed toxic metals and essential minerals in the hair of Arizona children with autism and associated conditions. Pica occurred with ASD in 32% of the children [33]. Brown et al., investigated blood lead concentrations (BLC) in children presenting with ASD and found that pica and developmental delay were not useful indicators of elevated BLC. Pica occurred with ASD in 13.7% of the children [36].

In a study exploring eating patterns in children with ASD, Ahumada et al., perceived pica as a concomitant disorder of food selectivity. In the group reporting pica, 4.2% of the sample reported xylophagia (consumption of paper and wood products) and 4.2% foliophagia (consumption of leaves), whereas 5,6% had unspecified pica [34]. Crayons, followed by soap, paper, feces, dirt and Play-Doh in equal occurrence, were the preferred non-food items in an American population of children with ASD and pica [40].

Likewise, a study from South India reported that crayons were the most ingested items, followed by rubber, paper, chalk and mud. Analysis of gastrointestinal manifestations and associated comorbidities in children with ASD, revealed a prevalence of 36,5% with pica [42].

In two studies from the “Study to Explore Early Development”, pica was strongly associated with ASD and with vomiting, diarrhoea and loose stools. Pica was more frequent in children with ASD with intellectual disabilities (ID), 28,1% than in children with ASD without ID (14.2%), while in the population group the prevalence was 3,5%. However, pica was not elevated in children with developmental disabilities with neither ID nor ASD characteristics (3,2%) [14].

Papini et al., looked at the prevalence and recurrence of pica behaviours in early childhood within the “Avon Longitudinal Study of parents and children” [41]. Pica was reported in 312 (3.1%) children at least twice in the study. A significant association was found between pica and autism at all five waves (*p* < 0.001) in the study. Pica was present in 12.5% of the children with autism at the age of 36 months and in 10.7% at the age of 115 months. The prevalence of pica with autism present was the highest at 77 months of age (13.6%) [41]. In spite of the strong association between autism and pica, most children with pica had not received an autism diagnosis [37].

### ARFID and ASD

Of the ten studies of comorbid ARFID and ASD (Table 2), six studies initially diagnosed patients with ARFID before further assessment revealed an ASD diagnosis. The frequency of comorbid ASD (Fig. 2) in these studies ranged from 7.7% [54] to 60% [51]. In addition, in more than half of the ARFID patients with comorbid ASD, the ASD diagnosis was initially undiagnosed [51].The lowest frequency was reported in a treatment study of Family Based Treatment for ARFID (FBT-ARFID) [54], and the highest was in the poor prognosis subgroup of ARFID patients in a retrospective study [51].

The only study that began with a cohort of children diagnosed with ASD, reported a prevalence of ARFID of 28% [50]. They examined a multi-ethnical group of children with ASD in a low-income area of Gothenburg, Sweden. Of the 46 children in the study, 35 exhibited feeding difficulties, and 25 of these met the criteria for paediatric feeding disorder (PFD) [55]. Thirteen of the children with PFD also met criteria for ARFID. The presence of persisting regulation problems, especially concerning eating, significantly increased the risk (RR = 2.11; 95% CI 1.32–3.38) for later feeding disorder. All children with ARFID displayed a lack of interest and avoidance based on sensory sensitivity. The onset age of feeding problems in ARFID occurred prior to clinical suspicion for ASD.

The ARFID drivers, sensory sensitivity and low interest in eating, were more commonly reported in children with ASD than in children without ASD [52]. The combination of these drivers was the most common and fear of negative consequences was the least common driver. The same pattern was found in a surveillance study of children and adolescents with ARFID attending secondary care [56]. In addition to comorbid ASD/ARFID, younger age at diagnosis of ARFID, being male and a high level of distress relating to eating/food, predicted that these children had more than one driver. Children with the subtypes fear of food and lack of interest, more often suffered from lower weight [56].

The most comprehensive study of ARFID and ASD compared three groups of clinically assessed children: ASD only, ASD with comorbid ARFID, and typically developing children (TDC) [46]. Children with ASD and ARFID demonstrated differences in social functioning, sensory processing, eating attitudes and quality of life compared to children with ASD only or TDC. Only oral sensory processing scores of the sensory profile significantly predicted ARFID in autistic children. Full scale IQ (FSIQ) was not a predictor but was reported to influence the presentation of autistic symptomatology and impact on adaptive behaviour. The FSIQ scores were higher overall in the ASD-no-ARFID group compared to the ASD-ARFID group [46]. The last two studies of comorbid ARFID and ASD were case series reporting on optic neuropathy with vitamin A deficiency [45] and decreased bone health [48].

### Rumination disorder and ASD

Rumination disorder was only mentioned comorbid to ASD in one study (Table 3) as part of exploring functional gastrointestinal diseases and sleep problems in children with ASD. They found that 38,1% of the 97 children with ASD, admitted to an outpatient child psychiatry clinic in Ankara, Turkey, had probable functional gastrointestinal diseases, of which three children (3.1%) (Fig. 2) were diagnosed with rumination disorder [53].

### Assessment tools

Assessment procedures for feeding disorders were heterogenous, with studies employing different standardized instruments in combination with clinical examination with or without dietetic consultation to establish DSM 5 diagnoses. Examples of assessment tools were: Eating Disorders in Youth Questionnaire, parents report, (P-EDY-Q) the Child Eating Behaviour Questionnaire (CEBQ), the Pica, ARFID and Rumination disorder Interview-ARFID-Questionnaire, self-report measure of the symptoms (PARDI-AR-Q), the Pica, ARFID and rumination disorder interview (PARDI), Child Behavior Checklist (CBCL), Brief Autism Mealtime Behavior Inventory (BAMBI), three-day food record, Checklist for Autism Spectrum Disorder (CAS) and Food Frequency Questionnaire (FFQ). In the study reporting Rumination disorder comorbid to ASD, the Rome III Diagnostic Questionnaire for Parent Report Form (QPGS-RIII), was used (Tables 1, 2 and 3).

### Eating disorder outcomes

Only two of the 23 studies thoroughly reported on deficiencies detected in blood tests in children with ASD and pica, ARFID and rumination disorders. In a case series two out of four male adolescents with ASD and ARFID, had deficiencies in vitamin A, B12, E and zinc. All four had calcium and vitamin D deficiency. Nutritional rickets secondary to vitamin D deficiency were diagnosed in one underweight and one overweight patient. Low bone mineral density was confirmed by DXA in two cases, including in one normal-weight boy with multiple fractures (femoral neck fracture and multiple thoracic vertebral body compression fractures) and in one overweight boy with vertebral fractures from jumping from low heights. All patients were adequately supplemented upon detection of the deficiencies and referred to intensive feeding therapy [48]. Across the included studies, micro nutritional supplements and oral nutritional supplements were commonly recommended and mostly associated with clinical and nutritional improvement [35, 47, 48, 50].

Irreversible optic neuropathy associated with vitamin A deficiency was reported in five paediatric patients with ARFID, three of whom had comorbid ASD and were aged six, eight and 16 years. Despite Vitamin A supplementation, vision prognosis remained poor [45].

Nine of the ten studies reporting on ASD and ARFID included data on weight and/or BMI. In three studies height, weight and BMI were reported to be lower in children with ASD and ARFID compared to ARFID alone [46, 50, 56]. Nevertheless, weight status within the ASD and ARFID group among participants ranged from underweight to overweight. In the study by Nygren et al., [50], 11/13 of children with ASD and ARFID had consulted a dietician and 9/13 had been prescribed oral nutritional supplements. Three of the children had been diagnosed with iron deficiency in need of treatment. Psychosocial impairment related to the feeding disorder was reported in 85% of the children [50], and comorbid ASD and ARFID was associated with greater social difficulties and lower social quality of life compared to ASD without ARFID [46].

### Treatment approaches

The literature search identified six studies addressing treatment or remission. Behavioural interventions on pica in children with ASD were associated with positive clinical outcomes [12, 39]. Remission of ARFID was observed in four of the 13 children at follow-up two years after receiving the ASD diagnosis [50]. In this study no specific treatment was described. In a Japanese study ASD was associated with poor outcome in children with ARFID [51].

FBT- ARFID [49, 54] led to weight gain in underweight young people with ARFID and autism (42% of the participants) [49].

Datta et al., found that FBT-ARFID was feasible for treating, retaining and obtaining clinical outcomes in both adolescents and in younger children with ASD, but considered weight improvement alone to be an insufficient metric to characterize remission or recovery from ARFID [54].

## Discussion

This systematic review identified substantial co-occurrence of pica and ARFID in children with ASD, while evidence regarding rumination disorder (RD) in ASD was limited due to the scarcity of available studies. The reasons for the high co-occurrence remain incompletely understood. Feeding and eating are complex processes involving and requiring motor skills, sensory processing, behavioural flexibility, tolerance of social demands and collaboration, all domains that may be challenging for some children with ASD. Altered oral sensitivity may contribute to atypical eating behaviours in both pica and ARFID, although potentially through different mechanisms. In pica, sensory-seeking behaviour has been proposed as one possible contributor, while nutritional deficiencies may also increase the risk of ingesting non-food substances. In ARFID, particularly in the sensory-sensitive presentations, children may prefer highly predictable foods and meals and show marked sensitivity to smell, taste, appearance, temperature and texture. Repetitive eating routines and avoidance of food-related demands may therefore overlap with sensory sensitivities, restricted and repetitive behaviours, and a preference for predictability commonly observed in ASD. These overlapping characteristics may partly explain the elevated co-occurrence, although the available evidence does not allow firm conclusions regarding causal mechanisms.

Importantly, malnutrition was reported among children with ASD despite normal weight or overweight, suggesting that weight status alone may not adequately reflect the presence or severity of the feeding and eating disorders.

Both pica and ARFID were reported with severe and irreversible somatic consequences supporting the need for guidelines and routines for screening for pica and ARFID in children with ASD. Overall, few studies were retrieved and very few reported data beyond co-occurrence, indicating a need for clinical studies on eating disorders in ASD, particularly for RD, where only one study of co-occurrence was found.

The frequency of the comorbid ASD with pica and ARFID varied considerably depending on the study population and level of care, and according to whether participants were primarily diagnosed with ASD or with an eating disorder, as well as the diagnostic tools applied (Tables 1, 2 and 3). Given the heterogeneity across study designs, a statistically appropriate comparison had to be replaced by a more rigorous narrative synthesis.

Pica was the most studied eating disorder with 12 studies addressing the co-occurrence of pica in children with ASD. Most studies found a rate of pica in ASD around 10–14%, while comorbid ID was associated with even higher rates of pica [14]. Although previous literature suggests pica can be associated with high morbidity and mortality [13], no life-threatening events were reported, only less serious consequences of pica, such as vomiting, diarrhoea and loose stool [38]. The absence of life-threatening events may partly reflect the inclusion of paediatric studies only, as children are generally more closely supervised. However, serious consequences of pica, including cases requiring surgery to survive, have been reported in single-case studies. Thus, restricting inclusion criteria to studies with at least three participants may have resulted in underestimation of the potential severity and urgent clinical risks associated with pica.

Detection of pica was generally not performed with standardized tools but mostly reported as a single item on the questionnaires. When diagnosing pica in young children with ASD and intellectual challenges it is important to take into consideration that the diagnostic criteria state that ingestion of non-food items should be inappropriate for the developmental level of the patient. Some children with ASD and/ or ID may not understand the differences between edible and inedible objects (Autism Speaks, [57]). Mayes and colleagues emphasized that because of the high prevalence of atypical eating behaviours in autism, young children referred to feeding clinics and older individuals referred to eating disorder clinics for treatment of their limited food preferences may have undiagnosed ASD [40]. The findings indicate that screening for pica should be part of the standard assessment in ASD, as it is both highly prevalent and highly treatable.

ARFID was the most broadly studied eating disorder, and the knowledge base is increasing rapidly with half of the studies included published in 2025 alone. Only one of the studies assessed the rate of ARFID in children with ASD [50], whereas the others focused on the presence of ASD in children with ARFID. All studies, however, found that ARFID and ASD were commonly co-occurring, indicating that the presence of either ASD or ARFID should entail screening for the other. The clinical presentation of ARFID was largely independent of sex, ASD symptom levels and general ability, with only oral sensory sensitivity predicting ARFID [46]. In contrast to anorexia nervosa, which shows a pronounced female predominance, ARFID seems to have a more balanced sex distribution [58]. Given the higher prevalence of ASD among males, the potential influence of ASD on the sex distribution of ARFID may warrant further consideration.

The sequence of diagnoses, ASD diagnosed before pica, while ARFID was diagnosed before ASD, may partly reflect differences in the developmental and clinical recognition of these conditions. The feeding problems in ARFID may start as early as during the first six months [50], about the time for weaning from breast milk or bottle to solids, whereas ASD may not be recognised until later in childhood, particularly in individuals with less overt autistic presentations. In contrast, the diagnosis of ASD preceding pica may reflect the developmental criteria for pica, as non-food mouthing and ingestion may be developmentally normative in younger children.

Age at presentation may also represent another important distinction, namely the one between anorexia nervosa and ARFID. Individuals with ARFID tend to present at a younger age than those with anorexia nervosa ([58]; Inoue 2021). The developmental timing may reflect the eating and feeding problems, restricted food repertoire and sensitivity issues emerger in early childhood in children with ASD, whereas anorexia nervosa presents during adolescence.

Children with ASD most frequently reported food-related sensory sensitivities and lack of interest as primary drivers of ARFID; however, they also exhibited combinations of all three drivers, similar to children without ASD [52]. Family therapy has a strong evidence-base for the treatment of eating disorders in children, including anorexia nervosa, as demonstrated by a recent review and meta-analysis [59]. Likewise, intervention studies indicated that FBT-ARFID was feasible and effective also for children with comorbid ASD [49, 54], while behaviour treatment was reported effective for treating pica in ASD [12, 39]. Intensive multidisciplinary intervention (IMI) has shown meaningful improvements in young children with ARFID [16], although its effectiveness in children with ARFID and comorbid ASD remains insufficiently studied. However, when comparing the safe food repertoire in individuals with ARFID to those with anorexia nervosa, different nutritional adequacy of these foods and the underlying patterns of restriction should be considered when developing the nutrition care plan. Given the differences in dietary restriction patterns and nutritional needs, a multidisciplinary approach is considered beneficial in the treatment of both anorexia nervosa and ARFID. FBT-ARFID was the only intervention studied and was found to have effect on weight restoration independent of ASD. There was no need to differentiate the assessment and treatment approach for ARFID depending on ASD traits such as restrictive and repetitive behaviours and interests and sensory sensitivities. As with ARFID in the absence of ASD, it was necessary for interventions to account for the individual ARFID drivers [49, 52, 54]. However, both intervention studies indicated that for children with ASD and ARFID, restoring weight may not be sufficient as malnutrition is common despite normal weight and growth [47].

The evidence base on ASD and RD was virtually non-existent with only one study [53]. The reasons for the limited research of ASD and RD remain unclear. However, RD may be underrecognized and understudied in children with ASD, potentially because symptoms may be misinterpreted as reflux or overlooked in the context of co-occurring eating disorders and gastrointestinal symptoms [60–62].

Furthermore, pica can be diagnosed comorbid to other diagnoses of feeding and eating disorders [1], including anorexia nervosa. However, we found that studies including children with ASD mostly addressed specific eating and feeding disorders in isolation. Greater awareness of comorbid eating disorders in children with ASD and research encompassing a broader range of eating and feeding disorders seems warranted to advance the evidence base in the field.

### Implication for future research and clinical practice

The frequency of comorbid ASD with pica and ARFID was generally high, demonstrating the need for a multidisciplinary diagnostic and therapeutic approach. Clinicians should screen for ASD in all children and adolescents with eating disorders and vice versa. Given the psychosocial impact, growth impairment, family burden, and potentially severe consequences of nutritional deficits in eating disorders, earlier emphasis of assessment and management of disordered eating in ASD is warranted. More knowledge is needed regarding the identification and management of comorbid ASD with ARFID, pica and rumination disorder. More knowledge is also needed to better understand the differences between the young patients with ASD with anorexia nervosa versus those with ASD comorbid to pica, ARFID or RD. Further investigation of the relationship between pica and selective and restrictive eating in children with ASD, is also warranted. Research may also help determine whether identifying autistic traits and ASD in adolescents with comorbid eating disorders, can facilitate more tailored treatment approaches and improve treatment outcomes.

### Strengths and limitations

We have followed the PRISMA guidelines and included both educational and health databases. We only included studies with established diagnoses of eating and feeding disorder. This restricted the period we investigated, excluding older relevant literature. Older literature may have contributed to more information on pica and rumination disorders especially, as these challenges have been described previously, albeit under different names. The diagnoses included have all been subjected to changes in the diagnostic criteria in both the ICD and the DSM. The concept of autism has, however, also changed dramatically in the last decades, so that previous knowledge would not necessarily apply to how we understand autism today. This was also the reason for limiting the study to the most recent diagnostic manuals. However, we acknowledge that when excluding single case studies and studies examining subthreshold ASD traits, we may have missed valuable complementary information. More broadly, the limited number of studies and the substantial heterogeneity across the studies, constrained the comparability and generalizability of the findings.

## Conclusion

Pica and ARFID appear to co-occur frequently with ASD, whereas the evidence regarding rumination disorder remains very limited. Substantial overlap in sensory, behavioural and restrictive features has been observed across ASD, pica and ARFID. Pica and ARFID tended to emerge at a younger age than anorexia nervosa, had a more balanced gender distribution and may occur across the weight spectrum. Importantly, normal weight or growth did not exclude clinically significant nutritional deficiencies. The possible severe nutritional, medical and psychosocial consequences associated with pica, ARFID and rumination disorder in children with ASD underscore the importance of early identification and individualized, multidisciplinary care. More consistent use of available standardized screening and diagnostic instruments may facilitate earlier identification and improve clinical outcome and healthy growth. However, substantial heterogeneity in diagnostic and assessment approaches, together with the limited number of available studies, restricts firm conclusions regarding clinical presentation and treatment. Further research is needed to improve assessment and clinical guidance, particularly regarding rumination disorder in children and adolescents with ASD.

## Supplementary Information

Below is the link to the electronic supplementary material.

Supplementary Material 1

## Data Availability

No datasets were generated or analysed during the current study.

## Abbreviations

C: Children
CC: Control children
M: Mothers
P: Parents ADHD: Attention Deficit Hyperactive Disorder
ASD: Autism spectrum disorder
BAMBI: Brief Autism Mealtime Behaviour Inventory
BLC: Blood lead concentration
BMI: body mass index
CBCL: Child Behaviour Checklist CASD: Checklist for autism spectrum disorder (feeding problems1/30 items with five subitems including eating inedibles)
DD: developmental delay
ED: eating disorder
FFQ: Food Frequency Questionnaire
FHQ: Feeding History Questionnaire by The Children´s Hospital of Philadelphia (2013)
GISSI-17: Gastrointestinal symptom inventory for ASD
ID: intellectual disability
