# Supplementary material for: Pica, avoidant restrictive food intake disorder and rumination disorder in children and adolescents with autism spectrum disorder: A systematic review
Source: J Eat Disord. 2026 Sep 14;14:226. doi: 10.1186/s40337-026-01772-x (PMC13617875; doi:10.1186/s40337-026-01772-x)
Supplement: Supplementary file 1 [file 40337_2026_1772_MOESM1_ESM.docx]

**S1 Search strategies and databases**

**Database: CINAHL**

Search History

S49	S37 OR S48

S48	S36 AND S47

S47	S38 OR S39 OR S40 OR S41 OR S42 OR S43 OR S44 OR S45 OR S46

S46	"teens"

S45	"teenage*"

S44	"youngster*"

S43	"adolescent"

S42	"youth"

S41	(MH "Adolescence+")

S40	"child"

S39	"children"

S38	(MH "Child+")

S37	S27 AND S35	Narrow by SubjectAge: - adolescent: 13-18 years

Narrow by SubjectAge: - child, preschool: 2-5 years

Narrow by SubjectAge: - child: 6-12 years

Narrow by SubjectAge: - all child

S36	S27 AND S35

S35	S28 OR S29 OR S30 OR S31 OR S32 OR S33 OR S34

S34	TX asd

S33	TX (autism* or autist*)

S32	(MH "Autism Spectrum Disorder")

S31	TX asperger*

S30	(MH "Asperger Syndrome")

S29	TX (pervasive development w1 (disorder* or syndrom*))

S28	(MH "Child Development Disorders, Pervasive+")

S27	S1 OR S2 OR S3 OR S4 OR S5 OR S6 OR S7 OR S8 OR S9 OR S10 OR S11 OR S12 OR S13 OR S14 OR S15 OR S16 OR S17 OR S18 OR S19 OR S20 OR S21 OR S22 OR S23 OR S24 OR S25 OR S26

S26	TI eating OR AB eating

S25	TX (eating w1 (problem* or difficult*))

S24	TX challeng* feeding behavio*

S23	TX sever* restrict* diet*

S22	TI feeding OR AB feeding

S21	TX "feeding-related behavio*"

S20	TX (feeding w1 (difficult* or problem*))

S19	TX "feeding difficult*"

S18	TX "food restrict*"

S17	TX "food aversion*"

S16	(MH "Food Aversions")

S15	TX "food selectivity"

S14	TX "food refus*"

S13	TX "nutritional deficienc*"

S12	(MH "Child Nutrition Disorders+")

S11	(MH "Malnutrition+")

S10	TX "feeding behavio*"

S9	TX merycism*

S8	TX ((rumination w1 (syndrom* or disorder*)))

S7	(MH "Feeding and Eating Disorders of Childhood+")

S6	TI pica OR AB pica

S5	(MH "Pica")

S4	TX food neophobi*

S3	"arfid"

S2	TX avoidant restrictive food intake disorder*

S1	(MH "Avoidant Restrictive Food Intake Disorder")

**Database: Cochrane**

#1	MeSH descriptor: [Avoidant Restrictive Food Intake Disorder] explode all trees

#2	avoidant restrictive food intake disorder

#3	arfid

#4	food neophobi*

#5	MeSH descriptor: [Pica] explode all trees

#6	pica

#7	MeSH descriptor: [Rumination Syndrome] explode all trees

#8	rumination syndrom*

#9	merycism*

#10	MeSH descriptor: [Feeding and Eating Disorders] explode all trees

#11	MeSH descriptor: [Feeding Behavior] this term only

#12	MeSH descriptor: [Malnutrition] explode all trees

#13	MeSH descriptor: [Nutrition Disorders] this term only

#14	food refus*

#15	food selectivity

#16	fussy eating

#17	food aversion*

#18	nutritional NEXT deficienc*

#19	food NEXT restrict*

#20	feeding NEAR/2 difficult*

#21	feeding NEAR/2 problem*

#22	feeding NEXT related NEXT behavio*

#23	(feeding):ti,ab,kw

#24	sever* NEXT restrict* NEXT diet*

#25	challenging NEXT feeding NEXT behavio*

#26	eating NEXT problems

#27	eating NEXT difficult*

#28	(eating):ti,ab,kw

#29	{OR #1-#28}

#30	MeSH descriptor: [Child Development Disorders, Pervasive] explode all trees

#31	("pervasive developmental disorder"):ti,ab,kw

#32	asperger*

#33	autism*

#34	autist*

#35	("ASD"):ti,ab,kw

#36	{OR #30-#35}

#37	#29 AND #36

#38	MeSH descriptor: [Adolescent] explode all trees

#39	MeSH descriptor: [Child] explode all trees

#40	adolescen*

#41	child

#42	children*

#43	youth

#44	youngsters

#45	teenage*

#46	teens

#47	{OR #38-#46}

#48	#37 AND #47

**Database: EMBASE**

1.	exp avoidant restrictive food intake disorder/

2.	avoidant restrictive food intake disorder*.mp.

3.	arfid.mp.

4.	food neophobi*.mp.

5.	exp pica/

6.	pica.tw.

7.	exp rumination syndrome/

8.	(rumination adj2 (disorder* or syndrom*)).mp.

9.	merycism*.af.

10.	feeding disorder/ or feeding disorder*.mp.

11.	eating disorder/

12.	eating disorder*.mp.

13.	feeding disorder/

14.	feeding disorder*.mp.

15.	feeding behavior/

16.	feeding behavior*.mp.

17.	feeding behaviour*.mp.

18.	malnutrition/

19.	nutritional disorder/

20.	nutrition* disorder*.mp.

21.	nutritional deficiency.mp.

22.	food refusal/

23.	food refusal/ or food refus*.mp.

24.	food selectivity.mp.

25.	food fussiness/

26.	fussy eating.mp.

27.	food aversion.mp. or food aversion/

28.	food restrict*.mp.

29.	feeding difficulty/ or feeding difficult*.mp.

30.	feeding problem*.mp.

31.	feeding-related behavio*.mp.

32.	feeding.tw.

33.	sever* restrict* diet.mp.

34.	challenging feeding behavio*.mp.

35.	eating problems.mp.

36.	eating difficult*.mp.

37.	eating.tw.

38.	1 or 2 or 3 or 4 or 5 or 6 or 7 or 8 or 9 or 10 or 11 or 12 or 13 or 14 or 15 or 16 or 17 or 18 or 19 or 20 or 21 or 22 or 23 or 24 or 25 or 26 or 27 or 28 or 29 or 30 or 31 or 32 or 33 or 34 or 35 or 36 or 37

39.	exp autism/

40.	autism*.mp.

41.	autist*.mp.

42.	exp Asperger syndrome/

43.	asperger*.mp.

44.	developmental disorder/

45.	pervasive developmental disorder*.mp.

46.	ASD.tw.

47.	39 or 40 or 41 or 42 or 43 or 44 or 45 or 46

48.	38 and 47

49.	limit 48 to (child or preschool child <1 to 6 years> or school child <7 to 12 years> or adolescent <13 to 17 years>)

50.	child/

51.	children*.tw.

52.	adolescent/

53.	adolecen*.tw.

54.	youth.mp.

55.	juvenile/

56.	youngster*.tw.

57.	teenage*.tw.

58.	teens.tw.

59.	50 or 51 or 52 or 53 or 54 or 55 or 56 or 57 or 58

60.	48 and 59

61.	49 or 60

**Database: MEDLINE (Ovid)**

1.	exp Avoidant Restrictive Food Intake Disorder/

2.	avoidant restrictive food intake disorder*.tw.

3.	arfid.mp.

4.	food neophobi*.mp.

5.	exp Pica/

6.	pica.tw.

7.	exp Rumination Syndrome/

8.	rumination syndrom*.tw.

9.	rumination disorder*.tw.

10.	merycism*.mp.

11.	"Feeding and Eating Disorders"/

12.	Feeding Behavior/

13.	exp Malnutrition/

14.	Nutrition Disorders/

15.	food refus*.tw.

16.	food selectivity.tw.

17.	fussy eating.tw.

18.	food aversion.tw.

19.	food restrict*.tw.

20.	nutritional deficiency.tw.

21.	feeding difficult*.tw.

22.	feeding problems.tw.

23.	feeding-related behavio*.tw.

24.	feeding.tw.

25.	sever* restrict* diet*.tw.

26.	challenging feeding behavio*.tw.

27.	eating problems.tw.

28.	eating difficult*.tw.

29.	eating.tw.

30.	1 or 2 or 3 or 4 or 5 or 6 or 7 or 8 or 9 or 10 or 11 or 13 or 14 or 15 or 16 or 17 or 18 or 20 or 21 or 25 or 26 or 27 or 28

31.	exp Child Development Disorders, Pervasive/

32.	pervasive developmental disorder*.tw.

33.	asperger*.tw.

34.	autism*.tw.

35.	autist*.tw.

36.	ASD.tw.

37.	31 or 32 or 33 or 34 or 35 or 36

38.	30 and 37

39.	limit 38 to "all child (0 to 18 years)"

40.	Adolescent/

41.	exp Child/

42.	children.tw.

43.	youth.tw.

44.	yongsters.tw.

45.	teenage*.tw.

46.	teens.tw.

47.	40 or 41 or 42 or 43 or 44 or 45 or 46

48.	38 and 47

49.	39 or 48

**Database: PsycINFO**

1.	exp "avoidant/restrictive food intake disorder"/

2.	avoidant restrictive food intake disorder*.mp.

3.	arfid.mp.

4.	food neophobi*.mp.

5.	exp Pica/

6.	pica.tw.

7.	(rumination adj2 (disorder* or syndrom*)).mp.

8.	exp "rumination (eating)"/

9.	merycism*.mp.

10.	feeding disorders/

11.	feeding disorder*.mp.

12.	Eating Disorders/

13.	eating disorder*.mp.

14.	feeding behavio*.mp.

15.	nutritional deficiencies/

16.	malnutrition.mp.

17.	nutrition* disorder*.mp.

18.	food refusal/

19.	food refus*.mp.

20.	food selectivity.mp.

21.	food avoidan*.mp.

22.	food fussiness.mp.

23.	fussy eating.mp.

24.	exp Food/ and exp Aversion/

25.	food aversi*.mp.

26.	food restriction*.mp.

27.	(feeding adj2 (difficult* or problem*)).mp.

28.	feeding-related behavio*.mp.

29.	feeding.tw.

30.	sever* restrict* diet.mp.

31.	challenging feeding behavio*.mp.

32.	(eating adj1 (problem* or difficult*)).mp.

33.	eating.tw.

34.	1 or 2 or 3 or 4 or 5 or 6 or 7 or 8 or 9 or 10 or 11 or 12 or 13 or 14 or 15 or 16 or 17 or 18 or 19 or 20 or 21 or 22 or 23 or 24 or 25 or 26 or 27 or 28 or 29 or 30 or 31 or 32 or 33

35.	exp autism spectrum disorders/

36.	(autism* or autist*).tw.

37.	asperger*.mp.

38.	asd.tw.

39.	(pervasiv* adj3 child development disorder*).mp.

40.	35 or 36 or 37 or 38 or 39

41.	34 and 40

42.	limit 41 to (100 childhood or 140 infancy <2 to 23 mo> or 160 preschool age or 180 school age or 200 adolescence )

43.	child.tw.

44.	children.tw.

45.	adolescen*.tw.

46.	youth.tw.

47.	youngster*.tw.

48.	teenage*.tw.

49.	teens.tw.

50.	43 or 44 or 45 or 46 or 47 or 48 or 49

51.	41 and 50

52.	42 or 51
